# Granulocyte contamination of separated blood mononuclear cells from spontaneously tumorous mice.

**DOI:** 10.1038/bjc.1980.315

**Published:** 1980-11

**Authors:** R. K. Kumar, A. W. Lykke


*Br. J. Cancer* (1980) **42**, 794

***Short Communication***

### GRANULOCYTE CONTAMINATION OF SEPARATED BLOOD MONONUCLEAR CELLS FROM SPONTANEOUSLY TUMOROUS MICE

R. K. KUMAR AND A. W. J. LYKKE

*From the School of Pathology, University of New South Wales, Sydney, Australia*

Received 9 May 1980 Accepted 14 August 1980

MANY *in vitro* immunological assays use separated peripheral-blood mononuclear cells obtained by density-dependent flotation on Ficoll–Hypaque. This technique, developed by Böyum (1968), yields a population of cells that morphologically appear to be almost entirely lymphocytes ($\geq 95\%$). Although it is well recognized that a significant proportion of these cells are in fact monocytes (Böyum, 1968; Zucker-Franklin, 1974) many workers nevertheless ignore this contamination and regard the separated cells as an essentially pure population of lymphocytes. However, in certain circumstances, this assumption may lead to dangerous misinterpretation. Currie *et al.* (1978) recently demonstrated that Ficoll–Hypaque-separated peripheral-blood mononuclear cells from human patients with advanced malignant disease were frequently contaminated with immature granulocytes. In a substantial number of their patients, "left-shifted" granulocytes were so numerous that lymphocytes were reduced to a minor subpopulation of the separated cells. They point out that many apparent functional abnormalities of lymphocytes from cancer patients might well be ascribed to gross contamination of the cell population being studied. We report similar findings in spontaneously tumorous SJL/J mice, and the use of multi-channel particle-size analysis to identify such contamination.

Inbred strain SJL/J mice spontaneously develop a malignant lymphoma that histopathologically resembles human Hodgkin's disease (Murphy, 1963). Primary tumours are frequently transplantable (Murphy, 1969) and may be maintained as *in vivo* lines. We have been studying the *in vitro* immunological response of separated peripheral-blood lymphocytes from tumorous SJL/J mice. Separated mononuclear cells were routinely obtained by conventional Ficoll–Hypaque flotation. Blood was collected by retro-orbital puncture and diluted 1 in 3 in Medium RPMI 1640. One ml of diluted blood was layered over 1·5 ml of Ficoll–Hypaque (Pharmacia Fine Chemicals, Sydney) and centrifuged at 20°C for 20 min at 400 *g*. Separated mononuclear cells were collected from the interface and washed twice in fresh medium. While performing cell counts with a Coulter counter, we noticed that separated cells from the blood of spontaneously tumorous animals consistently generated higher-amplitude pulses on the oscilloscope display of the counter than did cells from normal animals or animals bearing transplanted tumours. Since the amplitude of the pulse on the oscilloscope is proportional to the volume of the particle passing through the counter-tube orifice, this finding suggested the presence of significantly larger cells.

Confirmation of this observation was obtained by subjecting the cell suspensions to multi-channel particle-size analysis. A typical size distribution of normal separated peripheral-blood mononuclear cells is seen in Fig. 1. Separated cells from spontaneously tumorous animals have an entirely different size distribution: a typical distribution curve

Correspondence to: Dr R. K. Kumar, School of Pathology, The University of New South Wales, P.O. Box 1, Kensington, Australia, 2033.

Fig. 1.—Cell-size distribution of separated cells obtained from normal SJL/J mouse blood by Ficoll–Hypaque density flotation. Cells were counted on a Coulter Model ZF counter and pulse–height analysis was performed on a Coulter C-1000 Channelyzer coupled to an X–Y Recorder. The principal peak represents lymphocytes and the small secondary peak represents monocytes.

Fig. 2.—Cell size distribution analysis of separated cells from a spontaneously tumorous SJL/J mouse.

is shown in Fig. 2. The mode of this distribution is considerably higher and the range is also much wider ($21{\cdot}5 \pm 20$ as compared with $13{\cdot}5 \pm 12$ for the principal peak of normal cells). Similar abnormal size-distribution curves were obtained with separated cells from 6 spontaneously tumorous animals. In contrast, distribution curves from animals bearing transplanted tumours were identical to the normal cell-size distribution pattern.

In seeking an explanation for these results, we performed light microscopic examination of smears of separated cells from tumorous animals and age-matched normal animals. Separated cells were prepared individually from 6–8 animals in each group. Normal animals were aged 8–16 weeks and 52–66 weeks, corresponding to the ages of transplanted and spontaneously tumorous animals respectively. Animals developing spontaneous tumours were examined when palpably enlarged abdominal lymph nodes were detected. Transplanted animals were examined 3–4 weeks after i.p. injection of $5 \times 10^6$ tumorous lymph-node cells, when splenic and mesenteric lymph node enlargement was palpable, and the tumour load was comparable to that of spontaneously tumorous animals. All animals were subsequently autopsied and the presence of lymphoma confirmed by histopathological examination. Initially, we examined smears stained with Leishman's stain. However, since morphological examination fails to reveal many cells that are actually monocytes and, furthermore, because conventional morphological criteria are difficult to apply to Ficoll–Hypaque-separated cells we also examined smears by cytochemical techniques for precise cell identification. The staining methods of Yam *et al.* (1971) for nonspecific esterase (NSE) and chloroacetate esterase (CAE) were used; monocytes stain densely positive for NSE, while CAE is a specific stain for granulocytes. A minimum of 300 cells were counted in each stained smear.

Differential counts are recorded in the Table. Spontaneously tumorous animals show marked contamination of the separated cell population by granulocytes, many of which are mononuclear in configuration and are therefore not differentiable in Leishman-stained smears. The increase in granulocytes is statistically significant ($P \ll 0{\cdot}001$, *t* test). The percentage of cells with NSE activity is also

TABLE.—*Differential counts of Ficoll-Hypaque-separated cells from normal and tumorous SJL/J mice*

| | Normal (%) | Spontaneous tumour (%) | Transplanted tumour (%) |
|---|---|---|---|
| Leishman's stain | | | |
| Neutrophils | 1 (±1) | 39 (±6) | 2 (±1) |
| Lymphocytes | 94 (±2) | 44 (±5) | 92 (±3) |
| Monocytes | 5 (±2) | 12 (±2) | 6 (±2) |
| Eosinophils | 0 | 5 (±2) | 0 |
| Cytochemical stains | | | |
| Monocytes (NSE +ve) | 17 (±2) | 28 (±4) | 16 (±3) |
| Granulocytes (CAE +ve) | 2 (±1) | 46 (±4) | 6 (±2) |
| Lymphocytes (NSE and CAE −ve) | 81 (±3) | 26 (±8) | 78 (±4) |

Figures in parentheses denote standard deviations.

increased ($P < 0{\cdot}005$, $t$ test) and this may in part be accounted for by the presence of an increased number of eosinophils, which may also stain $NSE^+$. True lymphocytes (both $NSE^-$ and $CAE^-$) constituted a mere 26% of the separated cells. In contrast, the cell population obtained from animals bearing transplanted tumours was indistinguishable from the normal, indicating no contamination with immature granulocytes in the separated cells. The distribution of cell subpopulations in normal animals was not significantly affected by age. The spontaneously tumorous animals did not exhibit leucocytosis, and examination of peripheral-blood films revealed no evidence of leukaemia. However, as might be expected, the peripheral-blood differential leucocyte count of these animals showed a marked increase in the proportion of granulocytes when compared to normal animals (from a mean value of 7% to 72%). There was no evidence of a leucoerythroblastic anaemia, and autopsy examination of the tumorous animals revealed no lymphomatous infiltration of the marrow. Animals bearing transplanted neoplasms had a normal differential leucocyte count.

These findings have important implications for tumour immunologists. Firstly, they provide support for the contention that Ficoll–Hypaque-separated "peripheral blood mononuclear cells" from cancer patients may have marked immature-granulocyte contamination which might affect the validity of published reports of functional lymphocyte abnormalities (Currie *et al.*, 1978). Since the separation method is density-dependent, this contamination is unavoidable if the cells are isopycnic. Secondly, data from experimental studies of spontaneously tumorous animals may be subject to similar introduced error. Thirdly, at least in the experimental system we have studied, contamination by immature granulocytes does not occur in cells from animals bearing transplanted tumours. Thus, this variable does *not* appear to affect the validity of data from studies of *in vitro* immunological responses in these animals. Fourthly, multi-channel cell-size distribution analysis appears to provide a rapid and easy means of screening for granulocyte contamination, since lymphoid and myeloid cells are distinguishable on the basis of cell volume (England *et al.*, 1975).

This work was supported by a grant from the National Health and Medical Research Council of Australia. We thank Mr S. Watkins for expert technical assistance. Access to the Coulter C-1000. Channelyzer and related equipment was made possible through the courtesy of Coulter Electronics, Sydney.

REFERENCES

BÖYUM, A. (1968) Isolation of mononuclear cells and granulocytes from human blood. *Scand. J. Clin. Lab. Invest.*, **21** (Suppl. 97), 77.

CURRIE, G. A., HEDLEY, D. W., NYHOLM, R. E. & TAYLOR, S. A. (1978) Contamination of mononuclear cell suspensions obtained from cancer patients by the Böyum method. *Br. J. Cancer*, **38**, 555.

ENGLAND, J. M., BASHFORD, C. C., HEWER, M. G., HUGHES-JONES, N. C. & DOWN, M. C. (1975) Simple method for automating the differential leucocyte count. *Lancet*, i, 492.

MURPHY, E. D. (1963) SJL/J, a new inbred strain of mouse with a high, early incidence of reticulum cell neoplasms. *Proc. Am. Assoc. Cancer Res.*, **4**, 46.

MURPHY, E. D. (1969) Transplantation of Hodgkin's like reticulum cell neoplasms of strain SJL/J mice and results of tumour reinoculation. *J. Natl Cancer Inst.*, **42**, 797.

YAM, L. T., LI, C. Y. & CROSBY, W. H. (1971) Cytochemical identification of monocytes and granulocytes. *Am. J. Clin. Pathol.*, **55**, 283.

ZUCKER-FRANKLIN, D. (1974) The percentage of monocytes among "Mononuclear" cell fractions obtained from normal human blood. *J. Immunol.*, **112**, 234.


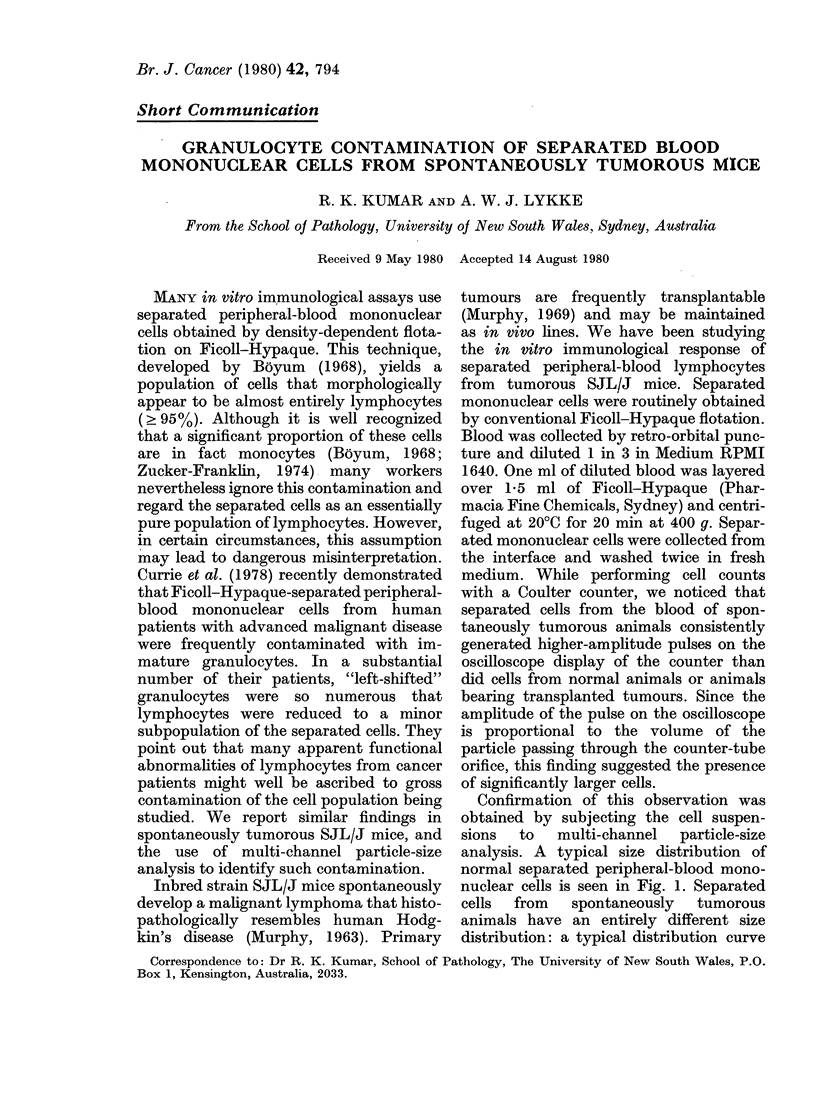

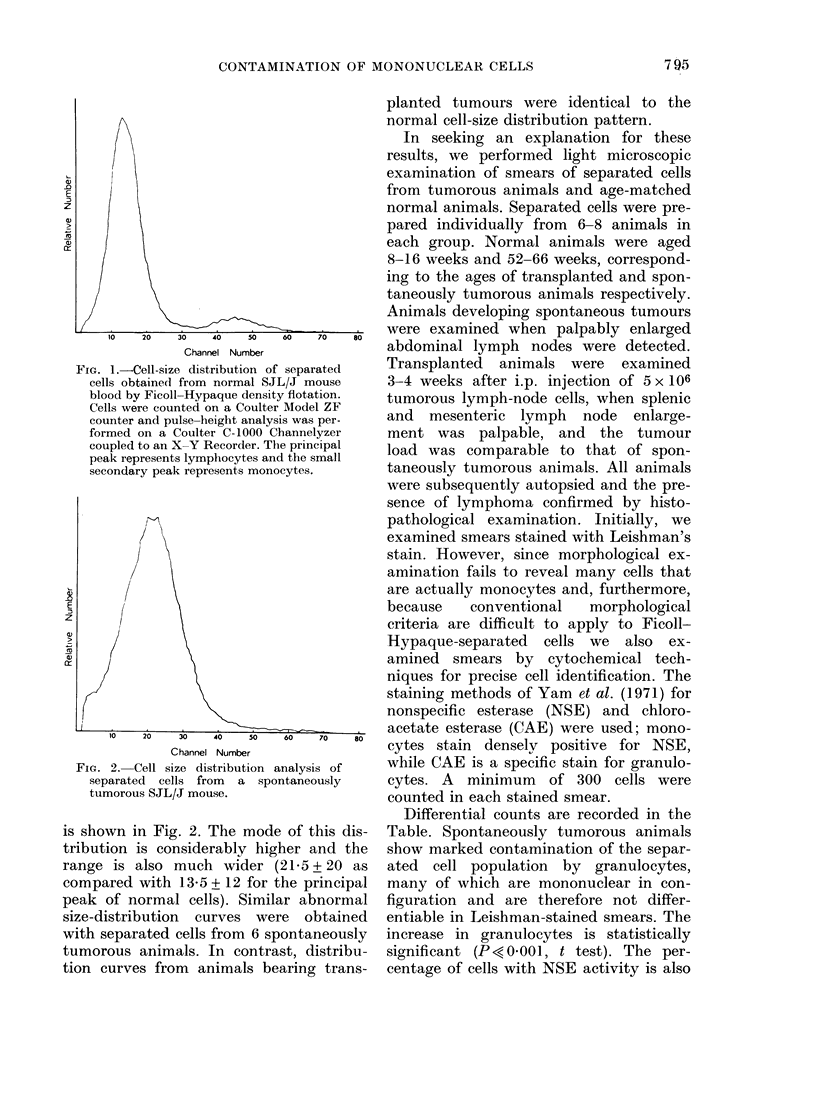

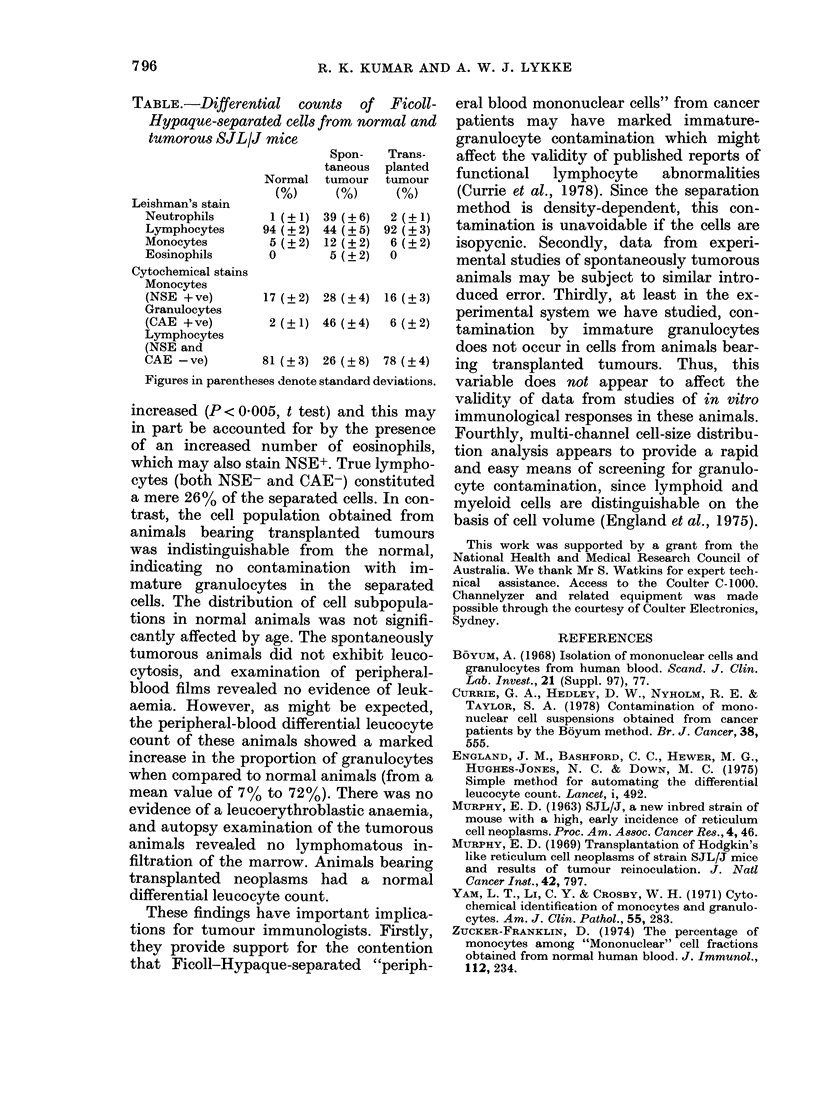


## References

[OCR_00305] Böyum A. (1968). Isolation of mononuclear cells and granulocytes from human blood. Isolation of monuclear cells by one centrifugation, and of granulocytes by combining centrifugation and sedimentation at 1 g.. Scand J Clin Lab Invest Suppl.

[OCR_00310] Currie G. A., Hedley D. W., Nyholm R. E., Taylor S. A. (1978). Contamination of mononuclear cell suspensions obtained from cancer patients by the Böyum method.. Br J Cancer.

[OCR_00317] England J. M., Hewer M. G., Bashford C. C., Hughes-Jones N. C., Down M. C. (1975). Simple method for automating the differential leucocyte-count.. Lancet.

[OCR_00327] Murphy E. D. (1969). Transplantation behavior of Hodgkin's-like reticulum cell neoplasms of strain SJL-J mice and results of tumor reinoculation.. J Natl Cancer Inst.

[OCR_00333] Yam L. T., Li C. Y., Crosby W. H. (1971). Cytochemical identification of monocytes and granulocytes.. Am J Clin Pathol.

[OCR_00338] Zucker-Franklin D. (1974). The percentage of monocytes among "mononuclear" cell fractions obtained from normal human blood.. J Immunol.

